# Exploring techniques for extraction of silver fir (*Abies alba*): phytochemical composition, antioxidant activity and cell viability

**DOI:** 10.1080/13880209.2025.2608481

**Published:** 2026-01-03

**Authors:** Katja Schoss, Urša Pečar Fonović, Nina Kočevar Glavač

**Affiliations:** Faculty of Pharmacy, University of Ljubljana, Ljubljana, Slovenia

**Keywords:** Antioxidant activity, silver fir (*Abies alba*), polyphenols, subcritical water extraction, cytotoxicity

## Abstract

**Context:**

Silver fir (*Abies alba*) contains polyphenols and lignans with antioxidant and therapeutic properties. Efficient extraction methods are essential to preserve these compounds and maximize bioactivity.

**Objective:**

To compare extraction techniques and identify the optimal method for obtaining high-quality silver fir extracts with strong antioxidant activity and minimal cytotoxicity.

**Materials & methods:**

Extracts from bark and branches were prepared using subcritical water extraction (SWE, 70–200 °C), supercritical CO_2_ extraction, and high-pressure ethanol extraction. Extracts were analyzed for total polyphenol content (TPC), lignan concentration (HPLC), antioxidant activity (DPPH and ABTS assays), and volatile profile (GC-MS). Cytotoxicity and cell migration were evaluated in HaCaT and Caco-2 cell lines *via* MTS and gap closure assays.

**Results:**

The SWE bark extract at 100 °C (SWE-BA-100) showed the highest TPC (73.8 mg GAE/g), lignan content (secoisolariciresinol 204.7 µg/mL), and antioxidant activity (DPPH: 24.2, ABTS: 32.0 mg GAE/g). Bark extracts had superior bioactive profiles compared to branches, though branch extractions gave higher yields. All extracts were non-cytotoxic. SWE-BA-100 inhibited cell migration, indicating a complex interaction between composition and cellular response.

**Discussion and Conclusion:**

SWE at 100 °C is a promising green method for producing potent antioxidant extracts from *A. alba*. Bark extracts offer strong antioxidant potential and safety for pharmaceutical, cosmetic, and nutritional uses. However, high lignan content may influence cellular behavior. Further studies should address the role of non-phenolic antioxidants and refine extraction strategies to balance efficacy and bioactivity.

## Introduction

The chemical composition and pharmacological effects of extracts from conifer species, including silver fir (*Abies alba* Mill.; Pinaceae), have attracted considerable attention in the fields of nutrition, cosmetics, pharmacy and medicine. Over 277 bioactive compounds have been identified in silver fir species, including a variety of polyphenols, terpenes and terpenoids that contribute to the plant’s protective functions and potential health benefits (Albanese et al. 2019).

Polyphenols, a large group of secondary metabolites, are crucial for plant defence against environmental stressors. There are about 8,000 known polyphenolic compounds that are divided into five main groups: flavonoids, lignans, stilbenes, polyphenolic acids and tannins (Pandey and Rizvi 2009; Daglia 2012; Barba, Esteve, and Frígola 2014). Silver fir extracts are particularly rich in flavonoids, lignans and phenolic acids, which have various bioactive properties, including antioxidant and antimicrobial properties (Kreft 2021).

The consumption of polyphenols has been shown to prevent the onset or development of various diseases related to oxidative stress (e.g., cardiovascular diseases, cancer, type 2 diabetes, osteoporosis and neurodegenerative disorders) (Yordi et al. 2012; Albanese et al. 2019; Hernández-Rodríguez et al. 2019). In silver fir, the highest concentrations of polyphenols are found in older branches and the bark. These polyphenols have a low molecular weight (flavonoids, lignans and phenolic acids) and play a role in protecting wood from pathogenic microorganisms, such as biocides, free radical scavengers and metal chelators, and in protecting a tree from degenerative processes such as the damaging effects of UV radiation (Vek et al. 2021).

Extracts of silver fir wood and bark with high polyphenol content have been shown to have anti-atherosclerotic (Drevenšek et al. 2015), cardioprotective (Drevenšek et al. 2016), anti-psoriatic (Starbek Zorko et al. 2018) and anti-diabetic effects (Debeljak et al. 2016; Lunder et al. 2019). They have also shown positive effects on osteoarthritis (Sirše et al. 2020). Recent research has discovered the extracts’ function as a prebiotic for bacteria of the genus *Lactobacillus* (Stojanov et al. 2021).

Given the therapeutic potential of silver fir’s polyphenols, efficient extraction processes are crucial to maximize their yield and preserve their bioactivity. Conventional extraction methods such as maceration and Soxhlet extraction are often limited by low efficiency and high energy consumption (Lomovsky et al. 2017). In response to the growing demand for more sustainable and efficient extraction processes, subcritical water extraction (SWE) has emerged as a promising alternative.

SWE is an environmentally friendly extraction technology that uses water in a subcritical state, i.e., at a temperature between 100 °C and 374 °C and under high pressure (usually 20 to 100 bar) to prevent boiling (Marcus 2018; Cheng et al. 2021). In its subcritical state, water has unique properties that make it an excellent solvent for the extraction of a wide range of bioactive compounds.

One of the main characteristics of subcritical water is its dielectric constant, which decreases when the temperature increases. This decreases the polarity of the water so that it is able to dissolve less polar organic compounds that are traditionally extracted using organic solvents such as ethanol or methanol. In addition, the lower viscosity and surface tension of subcritical water improve mass transfer, which increases the efficiency of the extraction of target compounds (Gbashi et al. 2017; Nastić et al. 2018; Zhang et al. 2020). Due to these properties, SWE can be used to efficiently extract polyphenols and other valuable phytochemicals from plant matrices, avoiding the environmental and health risks associated with organic solvents. However, the use of SWE also has disadvantages. High temperatures can lead to the decomposition of thermolabile compounds, and the equipment costs associated with maintaining the required pressures and temperatures can be significant (31). Although SWE has already been widely studied at the laboratory scale, its transfer to industrial production is still limited. Nevertheless, the use of water as the sole solvent, the absence of organic residues and compatibility with closed high-pressure systems make SWE highly attractive for pharmaceutical, cosmetic and food applications where product purity and regulatory compliance are essential. This indicates strong industrial potential with beneficial environmental impact, provided that further optimization and scale-up studies are undertaken. (Gbashi et al. 2017; Nastić et al. 2018; Zhang et al. 2020).

The application of SWE to silver fir and the comparison of the efficiency of different extraction methods have not yet been sufficiently explored. The aim of this study was to fill this gap by investigating the extraction of polyphenols from different parts of silver fir using SWE under different extraction conditions, and comparing the results with those of commercially available silver fir extract and extracts obtained using water, supercritical CO_2_ and ethanol under high-pressure to evaluate the potential of SWE as an environmentally friendly extraction method for industrial applications. We analyzed the composition of the extracts through HPLC and GC-MS, evaluated the total polyphenol content (TPC) using the Folin-Ciocalteu assay, and assessed the antioxidant activity using the DPPH and ABTS assays. The comparative analysis aimed to clarify the relationships between extraction conditions, yield, polyphenol content and antioxidant activity. Finally, the most promising extracts were analyzed for cytotoxicity using a cell viability assay (MTS) and tested for their effects on cell migration using a gap closure assay on Caco-2 and HaCaT cells.

## Material and methods

### Plant material

Plant material of silver fir (*Abies alba* Mill.) bark and branches was provided by AbiesLabs (Austria), a certified supplier. The plant material was authenticated based on supplier documentation and macroscopic/microscopic characteristics. As the material was received in an industrially processed form (dried and milled), the preparation of a classical herbarium voucher specimen was not possible. For traceability, an archival subsample of each batch (SNIL1810 for branches; CRMZ2011 for mixture of bark and branches) was retained.

Two types of raw plant material were used for extraction: 1) the industry standard, which consisted of silver fir branches (100%), and 2) test plant material, which consisted of silver fir bark (80%) and branches (20%). During the study, 12 samples of silver fir extracts were analyzed (extraction methods, parameters and abbreviations of the samples are summarized in Table 1). A commercially available extract of Belinal® (IND-BEL, lot: 191001) was used as a control sample; it was made using the industrial water extraction of silver fir branches at a ratio of plant material to water of 1:5 and a temperature of 70 to 90 °C for 30 to 60 min.

**Table 1.** List of extracts with extraction parameters and measured responses.

Abbreviation	Plant material	Extraction temperature (°C)	Pressure (bar)	Extraction process	Yield [%]	DPPH [mg/g]	ABTS [mg/g]	TPC [mg/g]	Isolariciresinol [𝜇g/ml]	Hydroxymatairesinol [𝜇g/ml]	Secoisolariciresinol [𝜇g/ml]	Lariciresinol [𝜇g/ml]	Pinoresinol [𝜇g/ml]	Matairesinol [𝜇g/ml]
SWE-BR-70	branches (100%)	70	100	Subcritical water extraction	0.5	10.3 ± 0.1	18.8 ± 0.0	55.0 ± 0.2	4.8	6.5	60.5	7.8	5.1	4.3
SWE-BR-100	branches (100%)	100	100	Subcritical water extraction	0.4	14.9 ± 0.1	22.3 ± 0.2	57.9 ± 0.1	13.0	13.2	115.4	17.0	12.2	9.5
SWE-BR-150	branches (100%)	150	100	Subcritical water extraction	1.0	4.7 ± 0.1	8.8 ± 0.1	29.9 ± 0.5	14.5	2.2	63.0	1.3	3.3	5.8
SWE-BR-200	branches (100%)	200	100	Subcritical water extraction	1.6	0.9 ± 0.1	5.1 ± 0.3	18.4 ± 0.3	7.9	<1	36.5	<1	<1	3.5
SWE-BA-70	branches (20%) + bark (80%)	70	100	Subcritical water extraction	0.2	21.4 ± 0.3	27.4 ± 0.0	61.9 ± 1.0	20.5	20.6	247.0	40.8	25.8	16.3
SWE-BA-100	branches (20%) + bark (80%)	100	100	Subcritical water extraction	0.2	24.2 ± 0.5	32.0 ± 0.1	73.8 ± 2.0	33.2	24.5	273.2	45.3	29.7	17.7
SWE-BA-150	branches (20%) + bark (80%)	150	100	Subcritical water extraction	0.4	20.9 ± 0.2	25.2 ± 0.1	51.4 ± 0.3	43.3	7.5	189.9	6.8	13.1	8.2
WE-BR-70	branches (100%)	70	1	Laboratory water extraction	0.5	14.0 ± 0.2	20.6 ± 0.2	51.0 ± 0.2	6.1	9.3	83.4	13.8	8.1	6.4
IND-BEL	branches (100%)	70	1	Water extraction on industrial scale	/	15.5 ± 0.1	18.3 ± 0.2	37.8 ± 0.9	26.7	13.1	112.3	9.0	12.3	12.5
SFE-CO2 CV1	branches (100%)	*30	*50	Supercritical CO2 extraction	/	0.7 ± 0.0	0.4 ± 0.0	0.2 ± 0.1	–	–	–	–	–	–
SFE-CO2 CV2	branches (100%)	*35	*75	Supercritical CO2 extraction	/	0.1 ± 0.0	0.3 ± 0.0	0.2 ± 0.0	–	–	–	–	–	–
HAE	branches (100%)	50	150	High-pressure EtOH extraction	2.1	8.7 ± 0.1	9.8 ± 0.0	10.2 ± 0.4	–	–	–	–	–	–

* Separation conditions of supercritical CO_2_ extraction in collection vessels.

Antioxidant activity and total amount of polyphenols, expressed as gallic acid equivalent in mg/g (mg GAE/g) of individual extract. The lignan concentrations are given in 𝜇g/mL, calculated from the calibration curves of the standards.

### Laboratory water extraction

The WE-BR-70 extraction procedure followed the basic industrial specifications used for Belinal® production (solid-to-liquid ratio 1:5, 70 °C, 60 min, filtration). As these are production requirements and detailed operational parameters from the manufacturer are not publicly available, the laboratory protocol used in this study is described below. Bark material was milled to a particle size of up to 5 mm and extraction was carried out using purified water at 70 °C for 60 min under constant magnetic stirring. The extract was then filtered through qualitative filter paper (Frisenette Aps, 12–15 μm pore size).

### Subcritical water extraction and supercritical fluid extraction

The SWE extracts were prepared in an SWE 5 L batch high-pressure extractor (Škrlj d.o.o., Slovenia), with a volume of five liters. An extraction pressure of 100 bar and a time of 60 min were constant for all extractions. Subcritical water was used to extract plant material consisting of 100% silver fir branches, and plant material consisting of silver fir bark (80%) and branches (20%). For the first sample type (branches), four extractions were performed at varying temperatures: 70 °C, 100 °C, 150 °C and 200 °C. For the second sample type (bark and branches), extractions were carried out at 70 °C, 100 °C and 150 °C. Purified water was used as a solvent with a ratio of plant material to water of 1:5. After extraction, approximately 5 liters of each aqueous extract were obtained, immediately filtered and stored in a freezer until further analysis.

SFE-CO_2_ extraction and high-pressure ethanol extraction (HAE) were performed in a MoSES 1.5.1 extraction unit (Škrlj d.o.o., Slovenia). The plant material (100% silver fir branches) was placed in a five-litre extraction vessel and the extraction process was carried out at a pressure of 200 bar and a temperature of 50 °C for 60 min. The supercritical CO_2_ and the extract mixture were separated in two collection vessels (CV1 and CV2) under the following separation conditions: sample SFE-CO2-CV1 at 30 °C and 50 bar (CV1), and sample SFE-CO2-CV2 at 35 °C and 75 bar (CV2). The extracts were stored in glass bottles at 4 °C until further analysis of the extracts.

HAE extraction was performed at 100 bar and 50 °C for 60 min. The mass ratio between ethanol and plant material was 5:1, the mass of the input material was 1,300 g. The obtained extract was dried using a rotary evaporator and stored at 4 °C until further analysis. The HAE sample differed from the others in terms of organoleptic properties, i.e., it was sticky.

### Antioxidant activity and total polyphenol content

The antioxidant activity of the extracts was evaluated using two complementary assays: the DPPH (2,2-diphenyl-1-picrylhydrazyl) and the ABTS (2,2′-azinobis-(3-ethylbenzthiazoline-6-sulfonic acid)) radical scavenging assays. ABTS and DPPH reagents were purchased from Sigma–Aldrich®; gallic acid, potassium peroxodisulfate and sodium carbonate (Na_2_CO_3_) were purchased from Fluka; ethanol was purchased from Carlo Erba; and Folin-Ciocalteu (FC) reagent was purchased from Merck. Spectrophotometric measurements were performed using a UV/Vis spectrophotometer (Nanocolor, Macherey-Nagel, Germany).

#### Sample preparation

For ABTS and DPPH assays, each extract was diluted in methanol to an approximate concentration of 1 mg/mL. The diluted samples were mixed using a vibratory mixer and sonicated for 15 min at room temperature using a sonicator. After sonication, the samples were filtered through 20 µm Frisenette filters and stored until analysis.

#### ABTS assay

The ABTS assay was performed according to Schoss et al. (Schoss et al. 2022), with minor modifications. Approximately 38 mg of ABTS was dissolved in 10 mL of purified water and 35 mg of potassium peroxodisulfate was dissolved in 50 mL of purified water. Both solutions were sonicated for five minutes and then 10 mL of the potassium peroxodisulfate solution was added to the ABTS solution. This mixture was incubated overnight at 4 °C protected from light, to allow the formation of the ABTS radical cation. Prior to the assay, the ABTS solution was diluted 40-fold with 96% ethanol to achieve an absorbance of 0.862 at 734 nm. For the assay, 100 µL of each extract was mixed with 900 µL of purified water, 1 mL of methanol and 8 mL of the diluted ABTS solution. The mixture was incubated for 6 min at room temperature, protected from light. The absorbance was then measured at 734 nm. All samples, including a blank control (without extract) and a negative control (without ABTS), were measured in triplicate. Antioxidant activity was expressed as mg gallic acid equivalents per gram of extract (mg GAE/g), with gallic acid standards prepared in methanol at concentrations ranging from 0.20 to 1.60 µg/mL. The calibration curve was fitted with the equation *y* = 417.5x + 0.0675 (R^2^ = 0.9943).

#### DPPH assay

The DPPH assay was performed according to Schoss et al. (Schoss et al. 2022), with minor modifications. A fresh DPPH solution was prepared by dissolving 80 mg DPPH in methanol to achieve a final concentration of 80 mg/L. For the assay, 2.5 mL of the DPPH solution were mixed with 100 µL of the extract, 900 µL of purified water and 1.5 mL of methanol. The mixture was then incubated for 30 min at room temperature under light protection on a shaker. After incubation, the absorbance of the samples was measured at 517 nm. Each sample was measured in triplicate, along with a blank control (without extract) and a negative control (without DPPH). Results were expressed as mg gallic acid equivalents per gram of dry extract (mg GAE/g). Gallic acid standards were prepared in methanol at concentrations ranging from 0.8 to 4.0 µg/mL, fitting a calibration curve with the equation *y* = 197.38x + 0.10610 (R^2^ = 0.9929).

#### Determination of total polyphenol content

The total polyphenol content (TPC) of the extracts was determined using the Folin-Ciocalteu (FC) spectrophotometric method according to the procedure described by Schoss et al. (Schoss et al. 2022), with minor modifications.

#### Sample preparation

Aqueous extracts were diluted with purified water to achieve a concentration of 1 mg/mL. For each sample, 100 µL were mixed with 4.9 mL of purified water and 300 µL of methanol. SFE-CO_2_ extracts were diluted in methanol to a concentration of 3 mg/mL, and 300 µL of each were mixed with 5 mL of purified water.

#### Reaction procedure

To each prepared sample, 300 µL of FC reagent were added, followed by a three-minute incubation. Then, 600 µL of a 20% Na_2_CO_3_ solution were added to the mixture. The samples were incubated for one hour on a shaker at room temperature, protected from light. Absorbance was measured at 750 nm. Each sample was analyzed in triplicate and a blank control (without extract) was prepared for comparison. TPC results were expressed as mg gallic acid equivalents per gram of dry extract (mg GAE/g). Gallic acid standards were prepared in water at concentrations ranging from 0.23 to 1.59 µg/mL. The calibration curve was fitted with the equation *y* = 471.58x + 0.0146 (R^2^ = 0.9997).

#### Statistical analysis

TPC, DPPH and ABTS assays were performed in triplicate. Pearson’s correlation (*p* < 0.05) and one-way ANOVA (95% CI, *p* < 0.01), followed by Tukey’s post hoc test, were performed using JASP 0.17.1.0 (University of Amsterdam, The Netherlands).

### High-performance liquid chromatography (HPLC) analysis of lignans

#### Sample preparation

Dried extracts were dissolved in 50% methanol to a concentration of 0.5 mg/mL for HPLC analysis, as previously described by Tavčar et al. (Tavčar et al. 2014). Samples were shaken and sonicated in an ultrasonic bath at 25 °C for 15 min, then centrifuged at 3,000 rpm for five minutes. The supernatant was filtered through 0.22 µm filters and transferred to HPLC vials.

#### HPLC conditions

HPLC-grade solvents were used throughout the analysis. Mobile phase A consisted of H_2_O, 2% acetonitrile and 0.1% trifluoroacetic acid, while mobile phase B consisted of acetonitrile, 2% H_2_O and 0.1% trifluoroacetic acid. Chromatographic separation was performed using a Shimadzu Prominence system equipped with a Phenomenex Kinetex® XB-C18 column (100 × 4.6 mm, 2.7 μm particle size). The gradient elution was as follows: 0–1 min − 5% B: 1–10 min − 5–30% B; and 10–15 min − 100% B. The flow rate was set at 2 mL/minute and the column temperature was maintained at 40 °C. Detection was performed using a photodiode array (PDA) detector set to record absorbance from 190 to 800 nm.

#### Quantification of lignans

Calibration curves were prepared for each lignan standard using five dilutions ranging from 10 to 50 µg/mL, except for secoisolariciresinol, where a range of 10 to 500 µg/mL was used. The concentration of each lignan (isolariciresinol, hydroxymatairesinol, lariciresinol, pinoresinol, secoisolariciresinol and matairesinol) in the extracts was calculated using the corresponding calibration curve, where y represents the peak height and x represents the concentration of the lignan (µg/mL). Linearity was established using R^2^ values exceeding 0.99 for all standards. The established calibration equations were as follows:

- **Isolariciresinol:** y = 957.55x + 835.1, R^2^ = 0.9995
- **Hydroxymatairesinol:** y = 472.86x + 673.4, R^2^ = 0.9933
- **Secoisolariciresinol:** y = 633.98x–4169.6, R^2^ = 0.9998
- **Lariciresinol:** y = 803.28x + 728.6, R^2^ = 0.9992
- **Pinoresinol:** y = 620.68x + 641.6, R^2^ = 0.9992
- **Matairesinol:** y = 518.9x + 15, R^2^ = 0.9999

The repeatability of sample preparation was assessed by preparing and analyzing three independent samples of extract SWE-BR-70. The relative standard deviation (RSD) of secoisolariciresinol responses was calculated, with an acceptance limit set at <5% RSD.

### Determination of volatile compounds

Hexane (GC-MS quality; J. T. Baker, US) extraction of the samples obtained using SWE was carried out at a ratio of hexane to sample = 1:20 and mixing on a shaker for one day. The hexane extract was analyzed through GC-MS using a method previously described by Kunc et al. (Kunc et al. 2022). Briefly, extracts were analyzed using a Shimadzu GC-MS system (GCMS-QP2010 Ultra. Japan) equipped with an MS detector and a Rxi-5Sil MS capillary column (Restek, USA; 30 *m* × 0.25 mm, film thickness 0.25 μm). The injector and ion source temperatures were set to 250 °C and 200 °C, respectively. The column temperature was programmed to increase from 40 °C to 270 °C at a rate of 3 °C/minute, holding the initial and final temperatures for 15 min. Samples of 1.0 μL were injected using an autosampler in split mode, with split ratios of 1:10. The MS detection was performed in electron ionization mode with an ionization energy of 70 eV, while the MS transfer line temperature was set to 250 °C. The mass-to-charge (m/z) range was from 40 to 400, with a scanning frequency of 5 Hz.

Compound identification was based on a comparison of the associated mass spectra and the retention indices with those of synthetic compounds in the National Institute of Standards and Technology (NIST20) and the Flavors and Fragrances of Natural and Synthetic Compounds (FFNSC4) spectral libraries. The linear retention indices were determined relative to a homologous series of n-alkanes (C6-C24). Compounds present at >1% relative peak area were reported. Unidentified compounds were annotated based on their three most intense fragment ions (m/z with relative intensity), where the first value represents the base peak (100%). No MS/MS fragmentation was performed. Method repeatability was verified prior to analysis, showing injection RSD values below 5%. Solvent blanks were analyzed between runs, and all hexane extracts were kept sealed and protected from light during the 24-h extraction to minimize oxidation or loss of volatiles. One representative GC-MS chromatogram is provided as Supplementary Material to illustrate the separation and spectral quality of the analyzed extracts.

### Determination of yield

The amount of extracted substances was determined through the freeze-drying (Lio 2000, Kambič, Slovenia) of the SWE and aqueous extracts applying programmed primary and secondary drying temperature of −20 °C and 20 °C (vacuum: 0.5012 mbar), respectively. The amount of ethanol extract was determined through drying in a rotary evaporator (R-200, B-490, V-710 BÜCHI, Switzerland) under reduced pressure at 60 °C, until constant mass was achieved. Extraction yield (%) was calculated as the mass of dry extract relative to the dry weight of the starting plant material. For SWE, 1 kg of dried and milled bark or branch material was used per batch with purified water as the only solvent. For other extraction methods, the same dry-mass-based calculation was applied. Moisture content of the plant material was not experimentally determined; therefore, yields are expressed relative to the nominal dry mass provided by the supplier.

### Cell cultures and treatment

Human keratinocyte (HaCaT, CRL-2404) and colorectal adenocarcinoma (Caco-2, HTB-37) cell lines, obtained from the American Type Culture Collection (ATCC), were cultured in Advanced DMEM (Gibco, US) supplemented with 10% fetal bovine serum (FBS), 1% L-glutamine and 1% penicillin-streptomycin (P/S) at 37 °C and 5% CO_2_, in a humidified atmosphere. Media were changed two to three times per week, and cells were subcultured weekly through trypsinization.

### Cell viability assay

Cell viability was determined using the MTS assay (CellTiter 96® Aqueous One Solution Cell Proliferation Assay, Promega, US). A total of 10^5^/mL of HaCaT cells and 7.5 × 10^4^/mL of Caco-2 cells were seeded in 96 well plates. Extracts were dissolved in PBS, sonicated at room temperature for 15 min, centrifuged at 3,000 rpm for 5 min and sterile filtered through a 0.22 µm filter (LLG Labware, Germany). Extract concentrations were set at 0.1 mg/mL or at 0.05 mg/mL for less soluble extracts (extracts SWE-BA-70 and IND-BEL). Cells were treated with extracts for 24 h, followed by the addition of 10 µL of MTS reagent to each well. Absorbance at 492 nm was measured after 1 h using a microplate reader (Tecan Safire, Tecan Group Ltd., Switzerland). The percentage of viable cells was calculated relative to the control cells with PBS, which were considered 100% viable. Two independent biological experiments were performed, each in quadruplicate.

### *In vitro* gap closure assay

To evaluate cell migration dynamics, a gap closure assay was performed. A total of 10^5^/mL of HaCaT cells and 1.5 × 10^5^/mL of Caco-2 cells were seeded into 6-well plates (TPP, Switzerland). Once a confluent monolayer was achieved, a wound was created using a 200 µL pipette tip. For HaCaT cells, a linear wound was created, while for Caco-2 cells the wound was circular. Cells were then treated with extracts at 0.05 mg/mL and further diluted in the culture medium (1:10 ratio). PBS with a culture medium (1:10 ratio) served as a control.

Images were taken at 0, 12, 18 and 24 h for HaCaT cells, while additional images at 39 h were taken for Caco-2 cells, using an EVOS^TM^ XL Core Imaging system (Thermo Fisher Scientific, US). Five locations on the cell-free created wound were selected for HaCaT cells, while whole circular wounds were imaged for Caco-2 cells. The wound area was measured using ImageJ software with a wound healing size tool (https://github.com/AlejandraArnedo/Wound-healing-size-tool/wiki) (Suarez-Arnedo et al. 2020), and the percentage of the open gap was calculated using the following formula, where S_0_ represents the gap area at time zero and S_t_ represents the average gap area at time t:

$$\text{\% open gap at time t }=\frac{\text{100 }\times \;S_{t}}{S_{0}}\;\left[\text{\%}\right]$$

## Results and discussion

### Determination of antioxidant activity, polyphenol content compounds and yield

Silver fir (*Abies alba*) is valued for its rich content of bioactive compounds, while efficient extraction techniques are essential for maximizing the yield and ensuring the quality of those compounds. This study compared different extraction techniques to optimize the industrial processing of silver fir extracts for diverse applications. For each sample, the extracted content expressed as yield in %, TPC content, and antioxidant activity determined using DPPH and ABTS assays expressed as gallic acid equivalent (mg GAE/g extract) were evaluated. The results are presented in Table 1.

The extracted compounds varied significantly among the samples, with the highest yield obtained using HAE extraction (2.1%; RSD = 1.1%). The highest yield of SWE branch extracts was comparable and reached 1.6% in sample SWE-BR-200. The yield was decreased by lowering the temperature. Comparable yields were observed for WE-BR-70 and SWE-BR-70, indicating no significant difference between laboratory water extraction and SWE at 70 °C (*p* > 0.05). In contrast, the yield of dry plant extract from branches was found to be about twice as high as that from bark at the same temperature. Overall, silver fir branch extracts demonstrated slightly higher dry matter yields than bark extracts.

The TPC, DPPH and ABTS assays showed positive correlations, with Pearson correlation coefficients of 0.56 (ABTS–DPPH, *p* = 0.001), 0.62 (ABTS–TPC, *p* < 0.001) and 0.86 (DPPH–TPC, *p* < 0.001). This is in line with previous studies, suggesting that the antioxidant activity of silver fir extracts is largely driven by polyphenolic compounds (Tavčar Benković et al. 2017).

The highest TPC was found in SWE-BA-100 (73.8 mg GAE/g), with other bark extracts (SWE-BA-70 and SWE-BA-150) showing slightly lower but not significantly different values (*p* > 0.05). Correspondingly, SWE-BA-100 also exhibited the highest antioxidant activity in both DPPH and ABTS assays (DPPH: 24.2 mg GAE/g, ABTS: 32.0 mg GAE/g). Branch SWE extracts showed lower antioxidant activity and TPC than the corresponding bark extracts. Considering their higher yield, this might indicate the significant presence of compounds with no or poor antioxidant activity. Among the branch extracts, SWE-BR-100 showed comparable antioxidant activity (DPPH: 14.9 mg GAE/g, ABTS: 22.3 mg GAE/g) to the industrial extract IND-BEL (DPPH: 15.5 mg GAE/g, ABTS: 18.3 mg GAE/g) and the laboratory water extract WE-BR-70 (DPPH: 14.0 mg GAE/g, ABTS: 20.6 mg GAE/g). The overall higher content of polyphenols in the bark than in the branches can also be explained by the protective function these substances have, i.e., for protection against pathogenic microorganisms and UV radiation. The lowest TPC and antioxidant activity were observed in SFE-CO_2_ extracts (CV1, CV2) due to the poor extraction efficiency of polyphenols in nonpolar solvents (Ameer et al. 2017). This confirms the critical role of polyphenols in the antioxidant activity of silver fir extracts.

The content of individual lignans in SWE, i.e., isolariciresinol, hydroxymatairesinol, secoisolariciresinol, lariciresinol, pinoresinol and matairesinol as dominant lignans in silver fir, revealed that the highest concentrations of secoisolariciresinol, the most abundant lignan in silver fir, were extracted at temperatures ranging from 70 to 150 °C. The lowest total lignan concentration was observed in SWE-BR-200, indicating that higher extraction temperatures reduce lignan content. The most favorable SWE extraction temperature for hydroxymatairesinol, secoisolariciresinol, lariciresinol, pinoresinol and matairesinol was between 70 and 100 °C, and significantly lower concentrations of these lignans were found at higher temperatures (i.e., 150 °C, 200 °C). An extraction temperature between 100 and 150 °C was optimal for isolariciresinol.

When increasing the temperature from 150 °C to 200 °C, higher yields but lower TPC and antioxidant activity was observed. This suggests that the number of additional extracted substances increases by increasing temperature, while the amount of total polyphenols and consequently antioxidant activity decreases, which means that major antioxidants have already been extracted at lower temperatures. To better illustrate this tradeoff between extract quantity and bioactivity, a combined yield-activity index (Yield × DPPH, Yield × ABTS, Yield × TPC) was calculated (Table 2). This index is not a statistical normalization, but an indicative parameter integrating extraction yield with antioxidant capacity per unit of starting biomass. (Yield*DPPH, Yield*ABTS, Yield*TPC, shown in Table 2).

**Table 2.** Extraction yield (%), total phenolic content (TPC), antioxidant activity (DPPH, ABTS), and calculated antioxidant indices (yield × DPPH, yield × ABTS, yield × TPC) of silver fir extracts.

	DPPH	ABTS	TPC	Yield	Yield*DPPH	Yield*ABTS	Yield*TPC
SWE-BR-70	10.3	18.8	55.0	0.53	5.4	9.9	29.2
SWE-BR-100	14.9	22.3	57.9	0.379	5.7	8.5	21.9
SWE-BR-150	4.7	8.8	29.9	0.969	4.5	8.5	29.0
SWE-BR-200	0.9	5.1	18.4	1.605	1.4	8.2	29.6
SWE-BR-70	21.4	27.4	61.9	0.244	5.2	6.7	15.1
SWE-BR-100	24.2	32.0	73.8	0.241	5.8	7.7	17.8
SWE-BR-150	20.9	25.2	51.4	0.435	9.1	11.0	22.4
WE-BR-70	14.0	20.6	51.0	0.534	7.5	11.0	27.2
HAE	8.7	9.8	10.2	2.08	18.1	20.5	21.2

These combined indices showed that although yields were higher, the antioxidant activity per amount of biomass processed did not proportionally increase between SWE-BR-150 and SWE-BR-200 or other extracts except HAE. HAE extracts demonstrated higher normalized antioxidant activity despite having TPC values comparable to those of SWE-BR-150 and SWE-BR-200. This observation indicates that non-phenolic constituents may also contribute to the antioxidant response in these extracts. In future studies, it would be valuable to characterize these compounds in more detail using LC-MS-based analytical approaches.

To summarize, our study revealed that SWE at 100 °C was the most optimal extraction method for polyphenols, with the highest antioxidant activity of the extracts. This finding is consistent with the research of Tavčar et al. (Tavčar et al. 2014), who also reported optimal extraction conditions at 100 °C for silver fir. Furthermore, bark-based plant material gave higher antioxidant activity and TPC, but lower yields, which emphasizes the importance of industrial optimization in terms of the proper selection and pre-preparation of silver fir raw material.

In addition to SWE, hydrodynamic cavitation (HC) has recently been reported as another water-based, solvent-free approach for recovering phenolic compounds from conifer biomass, such as spruce bark and silver fir twigs (Pozzo et al. 2025; Tienaho et al. 2025). Some studies suggest that HC can achieve comparable or even higher phenolic yields than conventional hot-water extraction under mild conditions; however, direct comparison with SWE results obtained in this study remains challenging due to differences in feedstock type, particle size and process parameters.

Although SWE is considered an environmentally friendly technique due to the use of water as the sole solvent, this study was conducted on a laboratory scale and did not assess energy consumption, solvent recovery or process economics. Therefore, statements on sustainability refer only to the extraction principle and not to industrial application. A comprehensive evaluation of large-scale feasibility, including energy demand, process integration and life-cycle or techno-economic assessment, remains a relevant direction for future work.

### GC-MS analysis

The results of the GC-MS analysis of the SWE silver fir extracts are presented in Table 3, with the relative peak area percentage of compounds above 1%. The volatile compounds were categorized into monoterpene hydrocarbons, oxygenated monoterpenes, sesquiterpene hydrocarbons, oxygenated sesquiterpenes, diterpene hydrocarbons, oxygenated diterpenes, other compounds and unknown compounds.

**Table 3.** Gas chromatography–mass spectrometry compositions of volatile compounds in SWE extracts according to their relative peak areas in %.

		RI		Branches (100%)				Bark (80%) + Branches (20%)		
Compound	Ret. Time	Ms	Db	SWE-BR-70	SWE-BR-100	SWE-BR-150	SWE-BR-200	SWE-BA-70	SWE-BA-100	SWE-BA-150
4-methyl-pentan-2-one	4.42	733	732	3.4	1.3	0.1	0.1	2.0	0.8	0.2
furfural	6.82	828	822	–	–	0.3	3.2	–	–	–
α-pinene	10.63	931	933	–	–	1.0	0.9	–	–	1.1
5-methyl-furfural	11.80	957	960	–	–	–	1.1	–	–	–
cymene (*para*)	14.76	1022	1025	–	–	0.1	1.5	–	–	–
limonene	14.99	1027	1030	–	0.4	0.4	1.3	0.4	–	0.3
terpinolene	17.69	1084	1086	–	–	0.1	1.3	–	–	–
93 (100), 108 (89), 95 (79)	17.88	–	–	–	–	0.4	1.2	–	0.2	0.4
α-campholenal	19.63	1124	1125	0.7	1.7	1.6	0.7	–	1.2	1.5
pinocarveol (*trans*)	20.33	1139	1141	3.0	4.5	2.1	0.5	1.1	2.7	2.8
camphor	20.58	1144	1149	14.7	12.3	4.1	1.3	6.4	5.3	3.1
91 (100), 79 (93), 94 (85)	20.83	–	–	–	1.4	1.1	–	–	0.9	1.3
pinocamphone (*trans*)	21.24	1158	1160	0.7	1.0	1.1	0.5	0.4	0.6	0.9
pinocarvone	21.33	1159	1164	1.3	1.7	1.0	0.3	0.6	1.3	1.3
borneol	21.84	1170	1173	2.3	3.9	3.5	1.7	0.9	2.3	3.1
pinocamphone (*cis*)	21.98	1173	1176	1.0	1.0	0.7	0.3	0.7	0.7	0.6
terpinen-4-ol	22.25	1179	1184	2.1	1.9	1.4	0.6	0.4	0.5	0.7
43 (100), 117 (82), 132 (78)	22.57	–	–	1.8	2.1	0.8	0.2	0.3	0.7	0.7
α-terpineol	22.95	1193	1195	11.4	11.6	19.3	7.5	2.4	3.4	6.6
verbenone	23.47	1205	1208	24.1	22.5	12.3	3.4	6.0	10.4	8.1
91 (100), 109 (90), 119 (61)	24.09	–	–	1.0	1.3	0.7	–	0.3	0.6	0.7
isoeugenol	34.25	1446	1452	–	–	–	1.3	–	–	–
epicubenol	42.57	1627	1631	–	–	–	1.1	–	–	–
132 (100), 135 (60), 91 (45)	44.56	–	–	0.5	0.7	1.1	–	–	–	–
134 (100), 107 (55), 79 (51)	47.18	–	–	–	–	0.6	3.0	1.8	1.8	2.0
123 (100), 57 (53), 85 (28)	47.48	–	–	–	–	0.2	–	0.8	0.9	1.3
83 (100), 105 (72), 119 (54)	50.59	–	–	5.6	6.5	3.7	1.4	11.8	12.1	8.3
83 (100), 105 (70), 119 (42)	51.52	–	–	–	–	0.8	0.6	–	0.2	2.0
juvibione	54.87	2012	2016	2.6	2.1	4.6	11.5	6.4	4.5	4.4
manool	56.10	2052	2062	2.7	1.1	2.4	–	0.6	0.4	0.6
dehydro-juvibione	57.02	2081	2091	11.5	11.7	19.4	35.9	30.0	21.9	21.3
43 (100), 134 (81), 107 (45)	58.72	–	–	4.6	5.2	6.6	–	16.4	15.4	16.9
83 (100), 55 (62), 95 (58)	58.99	–	–	–	–	1.0	–	1.5	1.6	1.7
83 (100), 55 (32), 69 (11)	59.14	–	–	–	–	0.3	–	3.1	2.6	2.7
monoterpene hydrocarbon				–	0.4	1.7	6.3	0.4	–	1.5
oxygenated monoterpene				64.1	65.2	50.5	23.2	19.0	29.5	30.0
sesquiterpene hydrocarbon				0.5	–	0.2	2.3	–	–	–
oxygenated sesquiterpene				14.1	13.8	24.5	49.9	36.4	26.4	25.9
diterpene hydrocarbon				–	–	–	0.3	–	–	–
oxygenated diterpene				2.7	1.1	2.4	0.3	0.6	0.4	0.6
other				3.4	2.0	0.8	4.9	2.9	1.5	0.9
unknown				15.2	17.5	20.0	12.9	40.8	42.1	41.1

The compounds were identified based on their mass spectra and retention indices (RI; Db, database RI; Ms, measured RI). Unidentified compounds are presented as numbers according to their three most intensive mass ion peaks.

GC-MS analysis revealed that the silver fir branch extracts were rich in monoterpenes, particularly at lower extraction temperatures. SWE-BR-100, extracted at 100 °C, exhibited the highest monoterpene content at 65.2%, followed closely by SWE-BR-150 at 64.6%. However, at 200 °C, the monoterpene content dropped significantly to 29.5%. This decrease is consistent with literature suggesting that high temperatures lead to the thermal degradation of volatile compounds (Jiménez-Carmona and Luque de Castro 1999; Gámiz-Gracia and Luque de Castro 2000; Çam et al. 2019).

The compounds with relative peak areas >5% were identified as major constituents. Among them, the most abundant monoterpenes were verbenone (3.4% to 24.1%), camphor (1.3% to 14.2%) and α-terpineol (6.6% to 22.4%). Notably, verbenone content peaked in SWE-BR-70 at 24.1% but significantly decreased to 3.4% in SWE-BR-200. Similarly, camphor showed a maximum concentration of 14.2% in SWE-BR-70, which dropped to 1.3% in SWE-BR-200. α-terpineol followed a similar pattern, with its highest content in SWE-BR-150 and a substantial decrease at 200 °C. This trend indicates that while lower temperatures (100 to 150 °C) favor the retention of these key monoterpenes, higher temperatures lead to their degradation. Consequently, SWE-BR-200 extracted at 200 °C not only exhibited the lowest monoterpene content but also a significant reduction in the most abundant monoterpenes.

As the extraction temperature increased, there was a notable rise in the content of oxygenated sesquiterpenes. SWE-BR-200 exhibited the highest oxygenated sesquiterpene content at 49.9%, a significant increase compared to SWE-BR-100 and SWE-BR-150, which contained 13.8% and 14.1%, respectively. Notably, the major sesquiterpenes, juvibione and dehydro-juvibione, increased with temperature, peaking at 35.9% and 11.5% in SWE-BR-200. This trend supports the hypothesis that higher temperatures favor the extraction of more complex and less volatile compounds.

In the silver fir bark SWE samples, a lower proportion of monoterpenes than in samples obtained from branches was detected. The most monoterpenes were detected in SWE-BA-150 with a total of 30.0%. The content of sesquiterpenes was highest in SWE-BA-70 (36.4%) and similar in samples SWE-BA-100 and SWE-BA-150 (26%). All three samples contained a very high percentage of unknown compounds (40.8–42.1%). Camphor (3.1–6.4%) and verbenone (6.0–10.4%) were dominant compounds in the monoterpene group, while juvibione (4.4–6.4%) and dehydro-juvibione (21.3–30.0%) were dominant in the sesquiterpene group.

Data from literature confirm that most parts of silver fir contain volatile compounds (essential oils) of similar but not identical composition, the most important compounds being monoterpene hydrocarbons, with smaller amounts of sesquiterpene hydrocarbons and only small proportions of oxygenated compounds (mostly monoterpenes and very few sesquiterpenes) (Ancuceanu et al. 2023). The needles are relatively rich in monoterpenes, with limonene (over 50% of the total content), α-pinene, β-pinene and camphene being the most abundant (Moukhtar et al. 2006; Duquesnoy et al. 2007). Silver fir bark and branches are not a typical plant material for the extraction of volatiles, so that a direct comparison of the results with literature is not possible. However, some studies have been carried out on the oleoresins of the bark, in which α-pinene (12.2–47.1%), β-pinene (14.9–36.6%) and limonene (5.5–41.4%) were predominant, although the composition varied greatly depending on the season (Zeneli et al. 2001).

In general, we determined that the most suitable temperature for the extraction of volatile terpenic compounds for both the SWE bark and SWE branch extracts is around 100 °C. For optimal extraction conditions, more detailed studies with a wider range of temperature variations should be performed. We concluded that a temperature of 200 °C drastically changes the volatile composition in the extracts, probably due to the thermal decomposition of the compounds.

### Viability of HaCaT cells and Caco-2 cells

The *in vitro* toxicity testing of extracts from herbal substances is extremely important as part of initial toxicological evaluation in order to ensure the proper selection of extract candidates that will later evolve to products that are safe for the user. Cytotoxicity was determined using the MTS assay on the HaCaT and Caco-2 cell lines. The HaCaT cell line is a spontaneously differentiated, immortalized keratinocyte line that is frequently used as a model to study various aspects of epidermal biology, including homeostasis and pathophysiology. The use of HaCaT cells provides insights into the differentiation and proliferation of keratinocytes, and their response to various stimuli. They are particularly useful in the study of skin diseases, wound healing and the effects of UV radiation or other environmental factors on the skin (Blanchard et al. 2022). The colorectal adenocarcinoma Caco-2 cell line is an important model for studying the intestinal barrier, absorption and transport. The cells are used for various drug and compound absorption assays, substance permeability assays and transcellular passage assays. Caco-2 cells are also used to study the interactions between food and the intestinal epithelium. They can be used to study molecular mechanisms that would be more difficult to access *in vivo* (Lea 2015).

**Figure 1.**
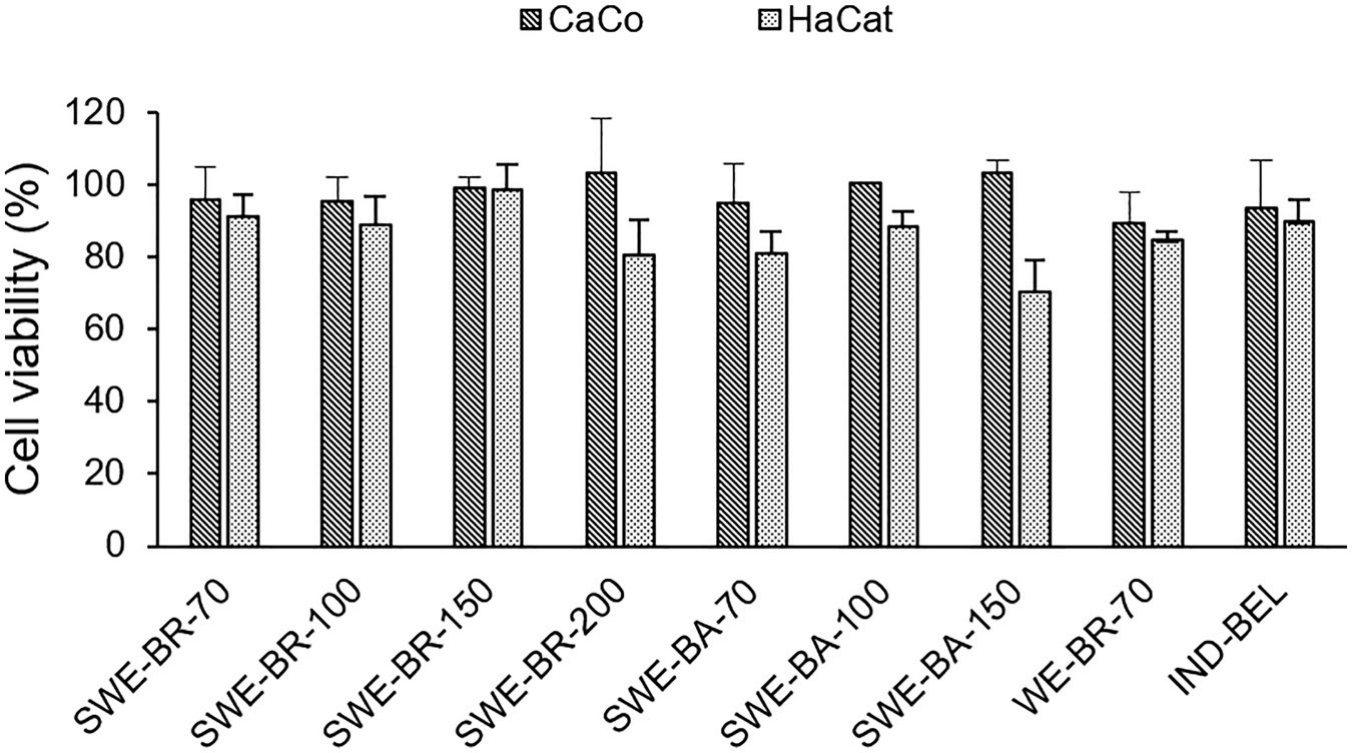
Viability of HaCaT and caco-2 cells after 24-hour exposure to the extracts. Data are presented as mean values ± RSD of two independent experiments, each performed in quadruplicate. The extracts SWE-BR-70, SWE-BR-100, SWE-BR-150, SWE-BA-100, SWE-BA-150, SWE-BR-200 and WE-BR-70 were tested at a concentration of 0.1 mg/mL, while the extracts IND-BEL and SWE-BA-70 were tested at a concentration of 0.05 mg/mL due to their poorer solubility.

According to the ISO 10993-5 standard, extracts are considered cytotoxic if they reduce cell viability by more than 30%, which means that cell viability is below 70% (International Organization for Standardization (ISO)) 2014). In our study, none of the tested extracts showed cytotoxic effects on HaCaT and Caco-2 cells, as all samples maintained cell viability above 70% after 24 h of incubation, as shown in Figure 1.

In HaCaT cells, the extracts SWE-BR-70, SWE-BR-150 and the industrial IND-BEL showed the highest viability rates (>90%), while the bark-based extracts and SWE-BR-200 showed slightly lower, but not statistically significant values (*p* < 0.05). In Caco-2 cells, the SWE-BA-100, SWE-BA-150 and SWE-BR-200 extracts also showed no significant variations from the control cells, and thus no cytotoxic effects.

No clear correlation was found between the content of total polyphenols, lignans or volatile compounds and cell viability, suggesting that the extraction method does not significantly affect cell survival. The results also showed no significant reduction in cell viability compared to the negative control, which is a positive sign for their potential use in medical, cosmetic or food applications. This assay was designed as a preliminary cytotoxicity screening using a single extract concentration in accordance with ISO 10993-5. Since all extracts maintained cell viability above 70%, no IC_50_ values could be calculated within this experimental design.

### Gap closure assay

**Figure 2.**
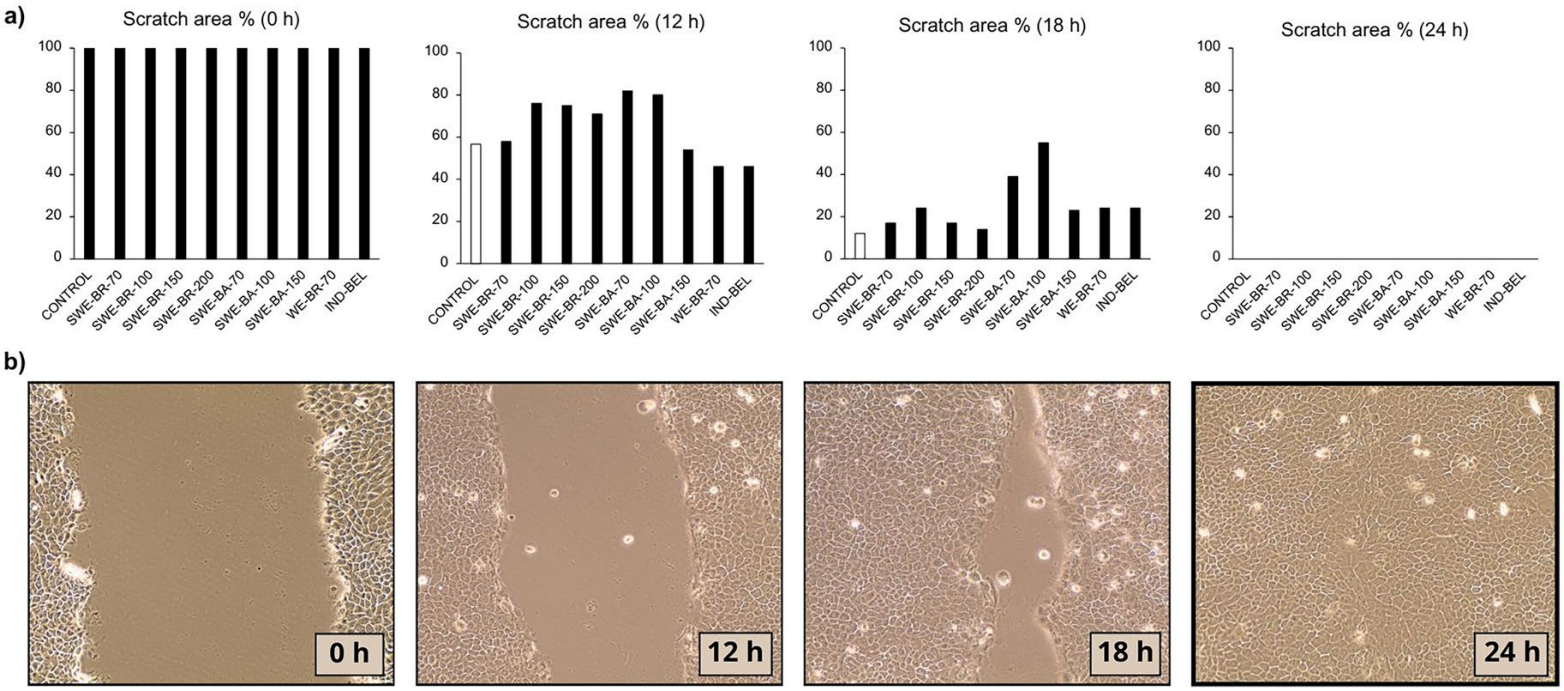
Gap area of HaCaT cells at time points 0 h, 12 h, 18 h and 24 h (*t* = 0 h was 100% of the gap area for all extracts, *t* = 24 h was 0% of the gap area for all extracts). a) the results represent the calculated value of the gap area as a percentage at different time points in relation to the initial gap at *t* = 0 for each extract. The different patterns on the columns represent individual samples: samples extracted from bark, samples extracted from branches, a control, the sample of the laboratory water extraction WE-BR-70 and the industrial extract IND-BEL. b) The progression of gap closure on HaCaT cells in the presence of extract SWE-BR-100 is shown.

**Figure 3.**
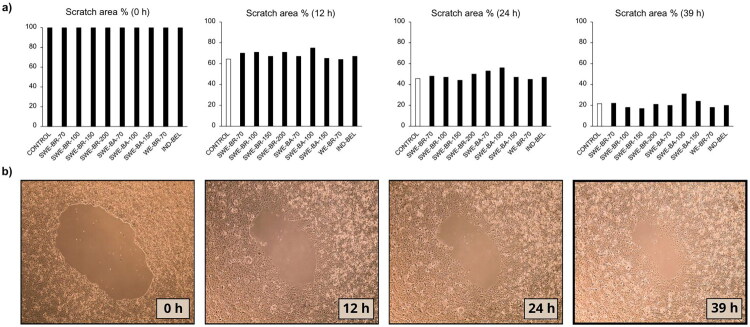
Gap area of Caco-2 cells at time points 0 h, 12 h, 24 h and 39 h (*t* = 0 h was 100% of the gap area for all extracts). a) the results represent the calculated value of the gap area as a percentage at different time points in relation to the initial gap at *t* = 0 for each extract. It is evident that the Caco-2 cells did not reach complete closure of the cleavage surface even after 39 h. The different patterns on the columns represent individual samples: samples extracted from bark, samples extracted from branches, a control, the sample of the laboratory water extraction WE-BR-70 and the industrial extract IND-BEL. b) The course of gap closure on Caco-2 cells in the presence of extract SWE-BR-100 is shown.

The gap closure assay is an *in vitro* method for determining cell migration, where the closure of an artificially created gap is observed, simulating renewal of damaged tissue. The gap closure assay showed that the extracts had different effects on cell migration, depending on their composition and preparation method. For both cell lines, the extract prepared using the conventional method (industrial water extraction) showed the strongest effect. The lignans contained in the extracts can influence cell migration, but the results showed that a higher concentration of lignans does not necessarily mean faster gap closure. This was the case with the extract SWE-BA-100, which had the highest total polyphenol content (TPC = 73.8 mg GAE/g), the highest lignan content (secoisolariciresinol = 204.7 µg/mL) and the highest antioxidant activity (DPPH: 24.2 mg GAE/g, ABTS: 32.0 mg GAE/g), while it inhibited cell migration in Caco-2 cells, with only 18% gap closure after 12 h. The other extracts obtained using the SWE method showed no significant effect on the gap-closure process. Especially in Caco-2 cells, the effect on migration was lower and the results were similar to those of the control. In HaCaT cells, complete closure of the open area occurred between 18 and 24 h, whereas in Caco-2 cells we did not achieve final gap closure even after 39 h, as shown in Figure 2. Representative images illustrating the progression of gap closure in HaCaT and Caco-2 cells after treatment with selected extracts are shown in Figure 3.

#### Influence of lignan content

The relationship between lignan content and gap closure was complex. While high lignan content, as in SWE-BA-100, was associated with the inhibition of migration, other extracts with moderate lignan contents, such as IND-BEL and SWE-BR-150, promoted migration or had no adverse effect on it. This suggests that the effect of lignans on cell migration may not be linear and could be influenced by other extract components or overall composition. The results are consistent with previous studies on polyphenol extracts from medicinal plants, which have been shown to promote wound healing by stimulating cell migration through different signaling pathways (Guo et al. 2021). However, our results suggest that excessive lignan content may counteract these benefits, possibly due to the pro-oxidant effects (Rajashekar 2023). Since only a single extract concentration was tested, these results should be interpreted with caution and cannot be directly attributed to lignans alone. Further studies using multiple concentrations and LC-MS-based profiling of lignan constituents would be useful to clarify their specific role in cell migration.

To summarize, silver fir extracts have different effects on cell migration depending on lignan content and extraction method. Extracts such as IND-BEL and WE-BR-70 were most efficacious in promoting early gap closure, making them potential candidates for applications in wound healing. In contrast, SWE-BA-100, with its high lignan content, consistently inhibited migration, highlighting the need for careful optimization of extract composition for therapeutic, cosmetic or nutritional applications. Since the migration assay was performed at a single extract concentration and without pharmacological migration controls, the results are indicative rather than mechanistic. Future dose-response experiments and real-time monitoring could help clarify lignan-related effects.

## Conclusions

This study demonstrated that silver fir (*Abies alba*) extracts, particularly those obtained from bark using subcritical water extraction at 100 °C, are rich in polyphenols and lignans, offering high antioxidant potential. The extraction method, temperature and silver fir part have a significant influence on the yield, polyphenol content and antioxidant activity. Extracts from bark proved superior to branch extracts, highlighting the bark’s protective role against damaging factors. While SWE efficiently extracts phenolic antioxidants, further research is needed to investigate non-phenolic antioxidants present in other extracts, in particular those from HAE. Moreover, the inhibitory effect of the lignan-rich SWE-BA-100 extract on Caco-2 cell migration indicates that extract composition can differentially modulate biological responses; however, as only a single concentration was tested, these findings should be interpreted with caution. Dose-response studies and LC-MS-based profiling of individual lignan and non-phenolic components would help to clarify their specific contribution to cell migration and wound-healing processes. Additionally, exploring the cytotoxicity and bioactivity of these extracts in more diverse cell models could help optimize their potential for therapeutic, cosmetic and nutritional applications, especially in wound healing and skin health.

## Supplementary Material

Supplement material 1.docx

## Data Availability

The data that support the findings of this study are available from the corresponding author upon reasonable request.
