# Supplementary material for: Exploring techniques for extraction of silver fir (Abies alba): phytochemical composition, antioxidant activity and cell viability
Source: Pharm Biol. 2026 Jan 3;64(1):168–84. doi: 10.1080/13880209.2025.2608481 (PMC12777867; doi:10.1080/13880209.2025.2608481)
Supplement: Supplement material 1.docx [file IPHB_A_2608481_SM7490.docx]

Supplementary Material 1.

Representative GC–MS chromatogram of the volatile fraction of the SWE-BR-100 extract (subcritical water extraction, 100 °C, branch material). Shown for documentation of separation and spectral quality. Peaks with >1% relative area are listed in Table 3.
